# Sesquiterpene Lactones from *Calea pinnatifida*: Absolute Configuration and Structural Requirements for Antitumor Activity

**DOI:** 10.3390/molecules25133005

**Published:** 2020-06-30

**Authors:** Lhaís Araújo Caldas, Mariana T. Rodrigues, Andrea N. L. Batista, João M. Batista, João H. G. Lago, Marcelo J. P. Ferreira, Ileana G. S. Rubio, Patricia Sartorelli

**Affiliations:** 1 Instituto de Ciências Ambientais, Químicas e Farmacêuticas, Universidade Federal de São Paulo, 09972-270 Diadema, SP, Brazil; lhais_araujo@hotmail.com (L.A.C.); ilerubio@gmail.com (I.G.S.R.); 2 Programa de Pós-Graduação em Biologia Estrutural e Funcional, Universidade Federal de São Paulo, 04021-001 São Paulo, SP, Brazil; mariana.rodr@outlook.com; 3 Instituto de Ciência e Tecnologia, Universidade Federal de São Paulo, São José dos Campos, 12231-280 São José dos Campos, SP, Brazil; andrluca@gmail.com; 4 Insitituto de Química, Universidade Federal Fluminense, 24020-141 Niterói, RJ, Brazil; 5 Centro de Ciências Naturais e Humanas, Universidade Federal do ABC, 09210-180 Santo André, SP, Brazil; joaohglago@gmail.com; 6 Departamento de Botânica, Instituto de Biociências, Universidade de São Paulo, 05508-090 São Paulo, SP, Brazil; marcelopena@ib.usp.br

**Keywords:** *Calea pinnatifida*, sesquiterpene lactones, vibrational circular dichroism (VCD), α,β-unsaturated carbonyl systems, thyroid carcinoma

## Abstract

This work describes the chromatographic fractionation of the aerial parts of *Calea pinnatifida* and the structural characterization and determination of the absolute configuration of the isolated compounds as well as their antitumor potential. The HPLC fractionation of the CH_2_Cl_2_ phase of the MeOH extract from the leaves of *C. pinnatifida* led to the isolation of two related sesquiterpene lactones (STLs): calein C (**1**) and calealactone B (**2**). Additionally, during the purification process, a derivative of calein C (**3**) was formed as a product of the Michael addition of MeOH. The structures of Compounds **1**–**3** were established based on spectroscopic and spectrometric data, while the absolute stereochemistry was established by vibrational circular dichroism. In order to evaluate the effect of the conjugated double bonds on the cytotoxic activity of STLs, Compounds **1**–**3** were tested against anaplastic (KTC-2) and papillary (TPC-1) thyroid carcinoma cells. Calein C was the most active of the STLs, and displayed activity against both KTC-2 and TPC-1. On the other hand, the calein C derivative (**3**) was the least cytotoxic of all the compounds tested. These results are promising and suggest the importance of studying sesquiterpene lactones isolated from *C. pinnatifida* in terms of antitumor activity, especially considering the effects of α,β-unsaturated carbonyl systems.

## 1. Introduction

*Calea pinnatifida* (R. Br) Less. (Asteraceae), popularly known as aruca, cipó cruz or quebra-tudo, occurs in Brazil, mainly in the “cerrado” biome [1]. This species is used in folk medicine as infusions to treat stomachaches, giardiasis, amoebiasis and gastric disorders in general [2]. According to the literature, phytochemical studies on the plant have revealed sesquiterpene lactones, fatty esters, steroids and a polyacetylene as the main components [2].

Sesquiterpene lactones (STLs) constitute a large group of secondary metabolites found in Asteraceae [3,4]. The presence of α,β-unsaturated carbonyl systems in STLs has been described as an important structural feature responsible for bioactivity, since they are capable of acting as Michael acceptors when in contact with biological nucleophiles [5,6]. Furthermore, Michael acceptor compounds are considered important alkylating agents, capable of supporting alkylation reactions through adequate nucleophiles [7].

According to the literature [3], germacranolides appear as one of the most frequent types of STL found in *Calea*, with more than 40 isolated compounds from *C. pinnatifida*, *C. ternifolia*, *C. urticifolia* and *C. zacatechichi* [8,9,10,11,12,13].

Despite the fact that sesquiterpene lactones from *Calea* have been isolated since the 1970s, important structural information about many STLs, such as their absolute configuration, is still unclear. This is due especially to the large number of possible substituents present in STLs, as well as the lack of specific techniques available in the past for solution-state assignments. Many of the experiments carried out on these molecules were based on X-ray crystallography and degradation reactions, as in the case of neurolenin B, a sesquiterpene lactone that had its relative configuration assigned based on X-ray crystallographic data [9]. Nowadays, bidimensional NMR experiments, combined with vibrational circular dichroism (VCD) measurements and calculations, represent powerful complementary tools to unambiguously define both the relative and absolute configurations of a given molecule directly in solution [14,15].

In terms of biological potential, literature data suggest that STLs might be responsible for the cytotoxic potential of many species, such as *C. pinnatifida* [16,17,18] and *C. urticifolia* [19,20]. Regarding antitumor activity, our research group showed that calein C, an STL isolated from *C. pinnatifida*, inhibits mitotic progression and induces apoptosis in MCF-7 cell lines by the inhibition of cell cycle progression at the M-phase [18]. Furthermore, analogues of calein C, such as arucanolide, displayed cytotoxicity against melanoma and HL60 cells [17,21].

Over the last years, the development of antineoplastic drugs based on natural products has been mainly due to combinatory chemistry, advanced spectroscopic and spectrometric instruments, and molecular networking [22,23]. However, many cancer tumors are resistant to some antineoplastic drugs administrated, not only the ones obtained from natural sources, but also the most common drugs used, such as cisplatin [24]. This scenario highlights the urgent need for prospecting new drugs.

Thyroid carcinoma is the fifth most common type of cancer among women worldwide [25]. The most common forms of thyroid cancer originate from thyroid follicular cells and can be divided into three major pathological forms: differentiated thyroid cancer (papillary thyroid carcinoma—PTC and follicular thyroid carcinoma—FTC) and undifferentiated thyroid cancer (anaplastic thyroid carcinoma—ATC).

According to literature data, natural products and plant extracts have demonstrated cytotoxic potential against thyroid carcinomas. For example, *Pulsatilla koreana*, a plant used in traditional Chinese and Korean medicines as an anti-inflammatory agent, showed cytotoxicity against ATC cell lines and caused a reduction in cell growth, inducing apoptosis [26,27,28].

Based on these issues, and also on the expressive bioactivity of related sesquiterpene lactones, this work aims to evaluate the cytotoxic potential of STLs isolated from *C. pinnatifida* (calein C, calealactone B and a derivative obtained from calein C) against thyroid tumor cell lines, exploring the differences observed between structures in terms of the presence of α,β-unsaturated carbonyl systems. Additionally the absolute configurations of natural products **1** and **2** were confirmed by VCD spectroscopy.

## 2. Results

### 2.1. Structural Elucidation of Compounds **1**–**3**

**Figure 1:**
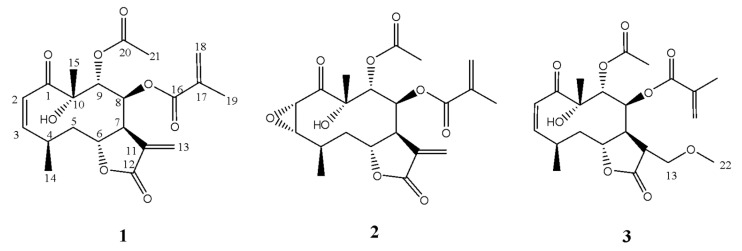
Structures of calein C (**1**), calealactone B (**2**) and the derivative of calein C (**3**).

The chromatographic procedures carried out allowed the isolation of three compounds: calein C (**1**), calealactone B (**2**) and a calein C derivative (**3**), a product of a Michael addition reaction with methanol (Figure 1).

**Figure 2:**
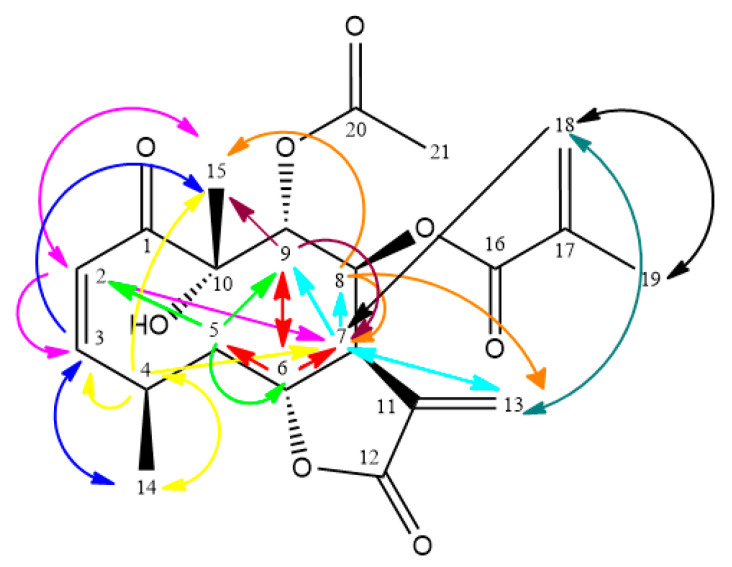
NMR NOESY correlations of **1**.

Compound **1** was obtained as a colourless crystal, mp. 170 °C. ESI-HRMS (positive mode) data displayed a *pseudo*-molecular ion peak at *m*/*z* 429.1542 [M + Na]^+^ corresponding to the molecular formula C_21_H_26_O_8_Na (calculated *m/z*: 429.1553). All signals in the ^1^H- and ^13^C-NMR spectra were attributed by the analysis of the ^1^H–^1^H COSY, HMQC, HMBC and NOESY (Figure 2) spectra recorded in benzene-*d*_6_.

Once the relative configuration of Compound **1** was unambiguously assigned by NOESY analysis, vibrational circular dichroism (VCD) experiments and calculations were used to determine its absolute stereochemistry for the first time in the literature. VCD associated with density functional theory (DFT) calculations has been demonstrated as a powerful probe for chirality in natural products [14,15].

**Figure 3:**
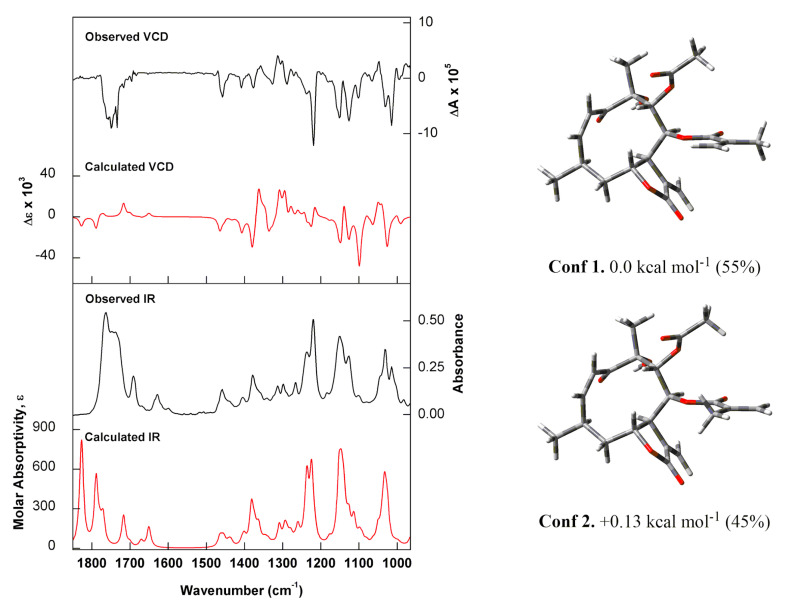
(Left) Comparison of experimental IR and vibrational circular dichroism (VCD) spectra of (−)-**1** recorded in CDCl_3_ (black trace) with the calculated [B3PW91/6-311G(d,p)] IR and VCD spectra for the Boltzmann average of the two lowest-energy conformers identified for (4*R*,6*R*,7*S*,8*S*,9*R*,10*R*)-**1** (red trace). (Right) Optimised structures, relative energies and Boltzmann populations of the lowest-energy conformers identified for (4*R*,6*R*,7*S*,8*S*,9*R*,10*R*)-**1**.

The very good agreement between the experimental VCD spectra obtained in CDCl_3_ and simulated data at the B3PW91/6-311G(d,p) level led to the assignment of the 4*R*,6*R*,7*S*,8*S*,9*R*,10*R* configuration to Compound **1** (Figure 3). The most representative vibrational modes used in this assignment were those at (−)-1025 cm^−1^ (C-O stretch of C-6, C-8, C-9 and C-10), (−)-1125 and (−)-1145 cm^−1^ (C-H bending of whole molecular framework), and (−)-1345 and (−)-1365 cm^−1^ (out-of-plane and in-plane C-H bending of C-6, C-7, C-8 and C-9).

Compound **1**, identified as calein C, was previously isolated from *C. urticifolia* [8] and *C. zacatechichi* [9]. Our group has also studied this compound previously in order to determine the correct relative position of the acetate and methacrylate groups, by means of HSQC and HMBC NMR in benzene-*d*_6_ (Table 1) [18,29], which were assigned to C-9 and C-8, respectively.

**Figure 4:**
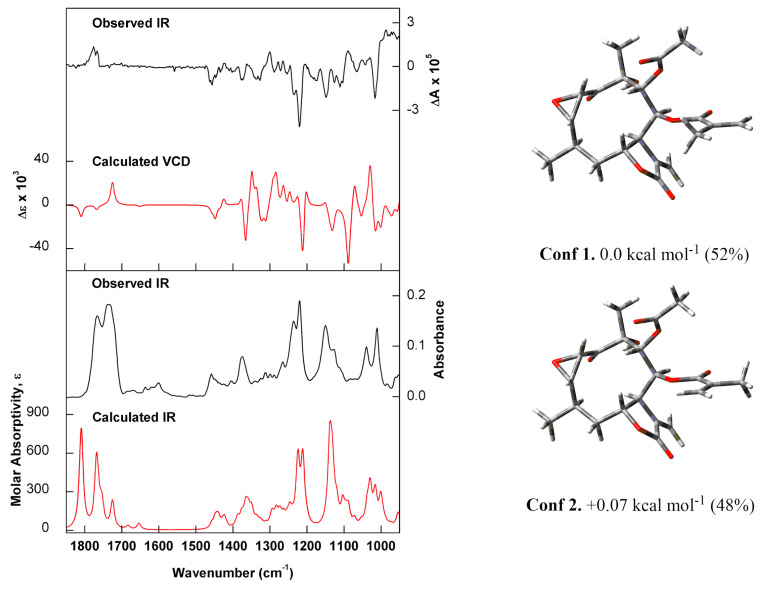
(Left) Comparison of the experimental IR and VCD spectra of (−)-**2** recorded in CDCl_3_ (black trace) with the calculated [B3PW91/6-311G(d,p)] IR and VCD spectra for the Boltzmann average of the two lowest-energy conformers identified for (2*S*,3*S*,4*R*,6*R*,7*S*,8*S*,9*R*,10*R*)-**2** (red trace). (Right) Optimised structures, relative energies and Boltzmann populations of the lowest-energy conformers identified for (2*S*,3*S*,4*R*,6*R*,7*S*,8*S*,9*R*,10*R*)-**2**.

NMR and MS data analyses of calealactone B (**2**) demonstrated that this STL is an analogue of calein C, containing an epoxide ring at C-2 and C-3. The ^1^H-NMR spectrum was similar to that of **1**, but it was possible to observe slight differences in H-2 (δ_H_ 4.23 *d*) and H-3 (δ_H_ 3.34 *dd*) patterns that correspond to oxirane hydrogens. The relative configuration of the stereogenic centers C-2, C-3, C-4, C-6, C-7, C-8, C-9 and C-10 in the structure of Compound **2** was established using the NOESY spectrum, and its absolute configuration was also unambiguously determined by means of VCD and DFT calculations. Comparisons of experimental and calculated spectra allowed the assignment of (−)-**2** as 2*S*,3*S*,4*R*,6*R*,7*S*,8*S*,9*R*,10*R* (Figure 4).

As expected, the VCD spectra obtained for (−)-**2** were very similar to those of (−)-**1**, as they share most of the structural and stereochemical features. The negative bands at around 1025, 1090 and 1140 cm^−1^, however, contain significant contributions from the C-H bending (out-of-plane) of C-2 and C-3, comprising the oxirane ring. The positive features between 1230 and 1260 cm^−1^ arise mainly from oxirane vibrations such as C-O stretch coupled to C-H bendings. Finally, the region between 1350 and 1400 cm^−1^ is influenced, among other vibrational modes, by in-plane C-H bendings of C-2 and C-3 coupled to C-C stretches to neighbouring carbons. Calealactone B was previously isolated from the genus *Calea* [11]. However, this compound has not been described in *C. pinnatifida*.

Compound (**3**), a calein C derivative obtained as an artefact by reaction with MeOH in Michael addition, was also evaluated. The HR-ESI-MS spectrum revealed the [M + H]^+^ peak at *m*/*z* 439.1980, corresponding to the molecular formula C_22_H_30_O_9_ (calculated for C_22_H_31_O_9_: *m*/*z* 439.1968). According to ^1^H-NMR data (Table 1), it was possible to observe signals at δ_H_ 3.36 (*s*, 3H) that suggested the presence of a methoxy group replacing the exocyclic double bond in the lactone ring. In the ^13^C-NMR, the presence of the signal δ_C_ 66.4 attributed to C-13, which appears shifted upfield compared to that observed for calein C (δ_C_ 126.3), indicated the absence of the exocyclic double bond at C-11/C-13 as present in the lactone ring of Compounds **1** and **2**. Additionally, the ^13^C data revealed the presence of an extra carbon δ_C_ 59.2, attributed to the methoxyl carbon. According to HMBC spectrum, it was possible to observe a correlation of H-13 (δ_H_ 3.70/3.35) with C-22 (δ_C_ 59.2) and C-12 (δ_C_ 174.1), as well as the correlations between H-7 (δ_H_ 3.08) and C-13 (δ_C_ 66.4), confirming the position of the methoxyl group at C-13 (see supplementary data).

In order to define the importance of the conjugated system in the lactone ring to the biological activity, Compound (**3**) was also investigated in terms of cytotoxicity against anaplastic and papillary thyroid cancer cell lines.

### 2.2. Biological Assays

Cytotoxic assays revealed that KTC-2 and TPC-1 tend to be more sensitive to calein C (**1**) than to the other substances tested (Table 2). This pattern is also observed in nontumor cells treated with (**1**) (NIH-3T3). Calealactone B (**2**) resulted in similar cytotoxic values against the cell lines tested when compared to calein C (**1**), probably due to their analogous chemical structures. The calein C derivative (**3**) was shown to be the least cytotoxic of the STLs tested in all cell lines, including fibroblasts.

In general, it is possible to observe that all substances were cytotoxic against all of the cell lines. However, the IC_50_ data show that calein C has a slight tendency to be more cytotoxic to the cells, mainly for papillary cells TPC-1 (IC_50_ 1.49 μM). In addition to that, anaplastic cells KTC-2 were also affected by the treatment with calein C (IC_50_ 1.67 μM). The literature also reports the cytotoxicity of calein C against the breast cancer cell lines MCF-7, MDA-MB-231 and Hs578T. The data suggest that calein C is able to reduce cell viability as well as inhibit cell cycle progression, consequently resulting in cell death [18].

Similarly to what is observed with calein C, calealactone B was also shown to be cytotoxic against both of the cell lines, whereas the IC_50_ values were slightly higher than those obtained for calein C. This difference might be due to the presence of an epoxide in positions C-2 and C-3 instead of the double bond present in calein C.

On the other hand, the cytotoxic potential of the calein C derivative (**3**) was the least effective against both cell lines tested, for anaplastic (IC_50_ 25.3 μM) and papillary (IC_50_ 23.6 μM). It was possible to see that the addition of the methoxy group at C-13, replacing the double bond, drastically changes the biological response.

The potential of a compound to be cytotoxic against anaplastic thyroid carcinoma is an interesting achievement considering that this type of carcinoma is highly aggressive and that no accurate treatment is currently available [30]. According to literature data, the cytotoxic potentials of isolated natural products against ATC are not widely studied. However, *Pulsatilla koreana* extract*,* used in traditional Chinese and Korean medicines, was shown to suppress the growth of anaplastic carcinoma in a dose-dependent manner, possibly also acting as an apoptosis inductor [26]. In addition, a flavonoid fraction obtained from *Citrus reticulata* juice also led to the reduction of ATC cell migration and proliferation in a time-dependent manner, acting in the G2/M phase of the cycle, and consequently promoting cell death [31].

Other phytochemicals, such as resveratrol, a phytoalexin present in several plant species; curcumin, a phenolic compound obtained from the rhizomes of turmeric (*Curcuma longa*); genistein, an isoflavone naturally found in numerous plants; and epigallocatechin gallate (EGCG), a polyphenolic compound found in green tea, induced a strong and significant reduction of the viability of ATC cell lines [32]. These results reinforce the importance of testing natural products on thyroid cancer cells with the objective of prospecting new drug prototypes.

According to the literature, the cytotoxicity of STLs containing an α,β unsaturated carbonyl system was demonstrated to be important when tested against *Trypanosoma brucei* [27]. Moreover, Padilla-Gonzalez and collaborators [5] described that α,β unsaturated carbonyl systems in STLs can participate in Michael addition with biological nucleophiles, showing high cytotoxic potential in biological media. Based on that, we believe that the presence of the α,β-unsaturated lactone found in Compounds **1** and **2**, might be important for the antitumor activity of STLs, since its loss in Compound **3** extinguished this activity.

## 3. Materials and Methods

### 3.1. General Experimental Procedures

^1^H- and ^13^C-NMR spectra of Compounds **1**–**3** were recorded, respectively, at 300 and 75 MHz in a Bruker Ultrashield 300 Advance III spectrometer. CDCl_3_ (Aldrich, St. Louis, MO, USA) and benzene-*d*_6_ (Aldrich) were used as solvents and as the internal standards. Silica gel *flash* (Merck, 230–400 mesh, New Jersey, NJ, USA) and Sephadex LH-20 (Amersham Biosciences, Little Chalfont, UK) were used for column chromatographic separations, while silica gel 60 PF_254_ (Merck) was used for analytical thin-layer chromatography (TLC). HPLC analysis was performed in a Dionex Ultimate 3000 chromatography system (Dionex, Sunnyvale, CA, USA), using a Luna Phenomenex RP-18 column (3 μm, 150 × 5 mm) and UV-DAD detector. IR and VCD spectra of Compounds **1** and **2** were recorded with a Single-PEM Chiral*IR*-2X FT-VCD spectrometer (BioTools, Inc., Jupiter, FL, USA) using a resolution of 4 cm^−1^ and a collection time of 6 h. The optimum retardation of the ZnSe photoelastic modulator (PEM) was set at 1400 cm^−1^. Minor instrumental baseline offsets were eliminated from the final VCD spectra of **1** and **2** by subtracting their VCD data from those obtained for the solvent under identical conditions. The IR and VCD spectra were recorded in a BaF_2_ cell with a 100 µm path length, using CDCl_3_ as the solvent. The samples were prepared as follows: 5 mg of **1** were added to 120 µL of CDCl_3_ and 3 mg of **2** were added to 110 µL of CDCl_3_.

### 3.2. Plant Material

Leaves of *Calea pinnatifida* (R. Br) Less. (Asteraceae) were collected from the Atlantic Forest area of São Paulo City, SP, Brazil (coordinates 23 53’08.86’’S, 46 40’10.45’’W), in October 2012. A voucher specimen (C.R. Figueiredo 25) has been deposited in the SPF Herbarium of Botany Department from the Biosciences Institute of University of São Paulo.

### 3.3. Extraction and Isolation of Compounds

Fresh leaves of *C. pinnatifida* (300 g) were dried, ground and then exhaustively extracted using MeOH at room temperature. After evaporation of the solvent under reduced pressure, the obtained crude extract (10 g) was resuspended in MeOH/H_2_O (2:1) and then partitioned with dichloromethane. The CH_2_Cl_2_ phase (650 mg) was subjected to column chromatography (CC) over Sephadex LH-20 using MeOH as a mobile phase to give seven fractions (A–G). Fraction B (420 mg) was subjected to CC over silica *flash* using increasing amounts of MeOH in CH_2_Cl_2_ as the solvent, to afford three fractions (B1–B3). Part of Fraction B1 (100 mg) was purified over semi-prep RP-18 HPLC, eluted with ACN/H_2_O (4:6) (flow rate: 3.6 mL min^−1^, λ: 218 nm), to afford calein C (**1**, 40.0 mg) and calealactone B (**2**, 9.0 mg). In the course of extraction and fractionation, a calein C derivative (**3,** 2.8 mg; 7.0% of **1**) was produced and isolated.

### 3.4. Cell Lines Culture

In order to assess the cytotoxic effects of the STLs, two thyroid cancer cell lines—KTC-2 (anaplastic thyroid cell line) and TPC-1 (papillary thyroid cancer cell line), donated by Professor Edna Kimura—and the murine fibroblast nontumor cell line NHT-3T3 were selected. KTC-2 and TPC-1 were maintained in RPMI-1640 (ThermoFisher Scientific, Waltham, MA, USA) supplemented with 5% and 10% fetal bovine serum (ThermoFisher Scientific), respectively, and 0.01 μg mL^−1^ of penicillin-streptomycin (ThermoFisher Scientific). NIH-3T3 was maintained in DMEM (ThermoFisher Scientific) supplemented with 10% fetal bovine serum (ThermoFisher Scientific). All cell lines were cultured at 37 °C with 5% CO_2_.

### 3.5. Cell Viability Assay and Determination of IC_50_ Value

The cell viability after drug treatments was determined with PrestoBlue Cell Viability Reagent (ThermoFisher Scientific), following the manufacturer’s instructions. Briefly, the thyroid cancer cells (KTC2 and TPC1) and the nontumor cell line (NIH-3T3) were seeded into 96-well plates at an initial density of 1000 cells per well and incubated at 37 °C for 48 h. Cells were treated with three concentrations of calein C (73.9, 36.9 and 18.5 μM), calein C derivative (68.3, 34.2 and 15.9 μM) and calealactone B (70.9, 35.4 and 17.7μM) for 72 h. A concentration of 10 μM of cisplatin was used as the positive control in the KTC2 and TPC1 cell lines for cytotoxicity. After 48 h of treatment, 10 μL of PrestoBlue reagent was added to each well and incubated for 1 h 30 min. Fluorescence (540 nm excitation/590 nm emission) was measured using a microplate reader, M3 (Molecular Devices, SoftMax Pro 7 Software). Vehicle-treated control cells (DMSO) were used to normalize the relative luminescence units from treated wells, and they were expressed as percentages of viable cells. The concentration to achieve 50% of cell death (IC_50_) was estimated from the non-linear regression analysis of the dose response curve using Prism 5 (GraphPad Prism 5, version 5.01). Cells were briefly treated with different concentrations of each compound for 72 h at concentrations ranging from 12.3 to 0.096 μM for calein C, 60.3 to 0.532 μM for calein C derivative and 17.7 to 0.137 μM for calealactone B. After 48 h treatment, 10 µL of PrestoBlue reagent was added to each well and incubated for 1.5 h. Fluorescence was also measured at M3. All experiments were performed in triplicate and data were expressed as the mean ± SEM.

### 3.6. Calculations

Calculations were performed for the arbitrarily chosen (4*R*,6*R*,7*S*,8*S*,9*R*,10*R*)-**1** and (2*S*,3*S*,4*R*,6*R*,7*S*,8*S*,9*R*,10*R*)-**2**. Conformational searches were carried out at the molecular mechanics level of theory with the Monte Carlo algorithm, employing the MM+ force field incorporated in the HyperChem 8.0.10 software package. Initially, 34 conformers of (4*R*,6*R*,7*S*,8*S*,9*R*,10*R*)-**1** and 29 conformers of (2*S*,3*S*,4*R*,6*R*,7*S*,8*S*,9*R*,10*R*)-**2** with a relative energy (rel E.) within 10 kcal mol^−1^ of the lowest-energy conformer were selected and further geometry-optimised at the B3LYP/6-31G(d) and B3PW91/6-311G(d,p) levels. The two conformers with rel E. < 4.0 kcal mol^−1^ for each compound, which corresponded to more than 99% of the total Boltzmann distributions, were selected for IR and VCD spectral calculations. DFT calculations were carried out at 298 K in the gas phase using Gaussian 09 software [33]. The IR and VCD spectral simulations were created using dipole and rotational strengths from Gaussian, which were calculated at the same level used during the geometry optimisation steps, and converted into molar absorptivities (M^−1^ cm^−1^). Vibrational analysis at the B3LYP/6-31G(d) and B3PW91/6-311G(d,p) levels resulted in no imaginary frequencies, confirming the considered conformers as real minima. Each spectrum was plotted as a sum of Lorentzian bands with half-widths at half-maximum of 6 cm^−1^. The calculated wavenumbers were multiplied by a scaling factor of 0.975. The final spectra were generated according to Boltzmann weighting of the lowest-energy conformers identified for **1** and **2** and plotted using the Origin 8 software.

## 4. Conclusions

From the MeOH extract of the leaves of *Calea pinnatifida*, two sesquiterpene lactones (STLs) were isolated: calein C (**1**) and calealactone B (**2**), while Compound **3** was obtained as a product of the Michael addition of MeOH to calein C. The absolute configurations of **1** and **2** were unambiguously confirmed as 4*R*,6*R*,7*S*,8*S*,9*R*,10*R* and 2*S*,3*S*,4*R*,6*R*,7*S*,8*S*,9*R*,10*R*, respectively, by means of VCD and DFT calculations. According to the biological data obtained, it was possible to observe that calein C, calealactone B and the calein C derivative were effectively cytotoxic against thyroid cancer cells, both papillary and anaplastic. However, differences in conjugated double bonds among the structures of the STLs were shown to influence their cytotoxic potential. KTC-2 and TPC-1 were shown to be more sensitive to calein C than to the other tested substances. Thus, the obtained results demonstrate how the presence of an α,β-unsaturated lactone ring might be important for the antitumor activity. To the best of our knowledge, this is the first report of the calein C derivative (**3**) and the structure–activity relationship among sesquiterpene lactone analogues from *C. pinnatifida*, suggesting the importance of conjugated systems in their biological potential.

## Figures and Tables

**Table 1:** ^1^H- (300 MHz) and ^13^C-NMR (75 MHz) spectral data for Compounds **1**–**3**.

		1 ^a^			2 ^b^		3 ^b^	
No	^1^H	^13^C	NOESY ^1^H		^13^C	NOESY	^1^H	^13^C
**1**	-	204.3	-	-	205.9	-	-	204.7
**2**	5.77 *d* (*J* 11.7 Hz)	124.9	H-15, H-7, H-3	4.24 *d* (*J* 4.2 Hz)	62.9	H-15, H-3, H-9	6.55 *d* (*J* 12.0 Hz)	125.6
**3**	4.72 *t* (*J* 11.7 Hz)	147.5	H-14, H-15, H-4, H-6, H-2, H-5	3.33 *dd* (*J* 9.3; 4.2 Hz)	55.6	H-2, H-14	6.01 *t* (*J* 12.0 Hz)	147.1
**4**	2.57 *m*	27.8	H-14, H-15 H-7, H-3	1.60 *sl*	26.0	OH, H-7	3.11 *m*	28.7
**5**	0.71 *m*/0.99 *m*	40.0	H-9, H-2, H-6	1.46 *m*/1.90 *m*	38.8	H-14, H-21	1.61 *s*/1.85 *brs*	38.2
**6**	4.13 *dd* (*J* 11.7; 4.8 Hz)	77.2	H-7, H-9, H-5	4.82 *dd* (*J* 11.9; 4.3 Hz)	79.7	H-5, H-6, H-8	4.62 *dd* (*J* 12.0; 4.1 Hz)	77.2
**7**	2.24 *brs*	41.3	H-6, H-8, H-9, H-13	2.36 *brs*	40.9	H-5, H-6, H-8	3.08 *m*	40.2
**8**	5.70 *dd* (*J* 9.8; 2.0 Hz)	74.6	H-7, H-13, H-15	5.68 *dd* (*J* 9.9; 1.2 Hz)	71.5	H-5, H-21, H-7, H-6	5.64 *m*	69.3
**9**	5.43 *d* (*J* 9.8 Hz)	73.9	H-6, H-7, H-15	5.77 *d* (*J* 9.9 Hz)	73.9	H-2, H-6, H-15	5.60 *d* (*J* 4.5 Hz)	74.7
**10**	-	79.1	-	-	74.6	-	-	79.2
**11**	-	131.5	-	-	134.3	-	-	37.5
**12**	-	168.0	-	-	168.3	-	-	174.1
**13**	5.02 *brs*/5.92 *brs*	126.3	H-7, H-18	5.83 *s*/6.33 *s*	126.8	H-7	3.35 *s*/3.70 *m*	66.4
**14**	0.33 *d* (*J* 6.3 Hz)	19.0	H-4, H-3	1.22 *d* (*J* 6.1 Hz)	18.6	H-3	1.16 *d* (*J* 6.0 Hz)	19.8
**15**	0.84 *s*	23.2	H-2, H-9	1.46 *s*	24.5	H-2	1.32 *s*	23.5
**16**	-	165.2	-	-	165.3	-	-	165.4
**17**	-	135.7	-	-	134.8	-	-	131.5
**18**	4.93 *s*/5.96 *s*	125.2	H-7, H-13, H-19	5.57 *brs*/6.04 *brs*	127.3	H-19	5.57 *s*/6.05 *s*	127.5
**19**	1.58 *s*	17.8	H-18	1.85 *s*	18.1	H-18	1.86 *s*	18.0
**20**	-	170.0	-	-	170.4	-	-	170.1
**21**	1.38 *s*	19.5	-	2.05 *s*	20.3	H-9, H-19	2.02 *s*	20.4
**22**	-	-	-	-	-	-	3.36 *s*	59.2

^a^ Benzene *d*_6_; ^b^ CDCl_3._

**Table 2:** IC_50_ (µM) determined for anaplastic cancer cells (KTC-2), papillary cancer cells (TPC-1) and murine fibroblasts (NIH-3T3).

	KTC-2	TPC-1	NIH-3T3
Calein C (**1**)	1.67	1.49	3.06
Calealactone B (**2**)	4.69	3.54	4.40
Calein C derivative (**3**)	25.32	23.25	18.54
Cisplatin	2.21	3.05	10.38

IC_50_ values were calculated using non-linear regression in GraphPad Prism 5.0.

## Supplementary Materials

The following are available online, Figure S1: ^1^H-NMR spectrum of (**1**), Figure S2: ^13^C-NMR spectrum of (**1**), Figure S3: HMBC NMR spectrum of (**1**), Figure S4: NOESY NMR spectrum of (**1**), Figure S5: ^1^H-NMR spectrum of (**2**), Figure S6: ^13^C-NMR spectrum of (**2**), Figure S7: NOESY NMR spectrum of (**2**), Figure S8: ^1^H-NMR spectrum of (**3**), Figure S9: ^13^C-NMR spectrum of (**3**).
