# Supplementary material for: Sesquiterpene Lactones from Calea pinnatifida: Absolute Configuration and Structural Requirements for Antitumor Activity
Source: Molecules. 2020 Jun 30;25(13):3005. doi: 10.3390/molecules25133005 (PMC7411797; doi:10.3390/molecules25133005)
Supplement: Supplementary file 1 [file molecules-25-03005-s001.pdf]

Sesquiterpene lactones from *Calea pinnatifida*: absolute configuration and structural requirements for antitumor activity

Lhaís A. Caldas[1], Mariana T. Rodrigues[2], Andrea N. L. Batista[3], João M. Batista Jr.[4], João H.G. Lago[5], Marcelo J. P. Ferreira[6], Ileana G. S. Rubio[1,2], Patrícia Sartorelli[1]*.

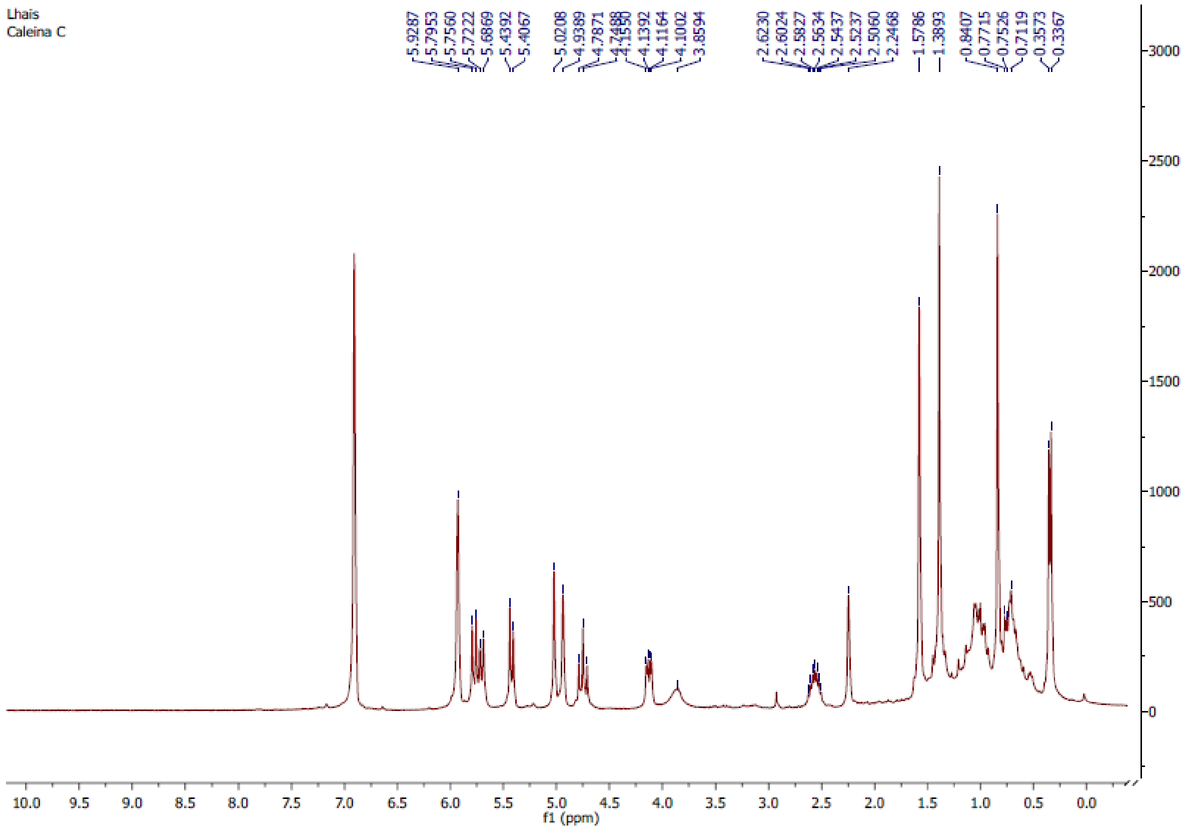

Figure S1: ^{1}H NMR spectrum of **(1)** (300 MHz; Benzene d_6)

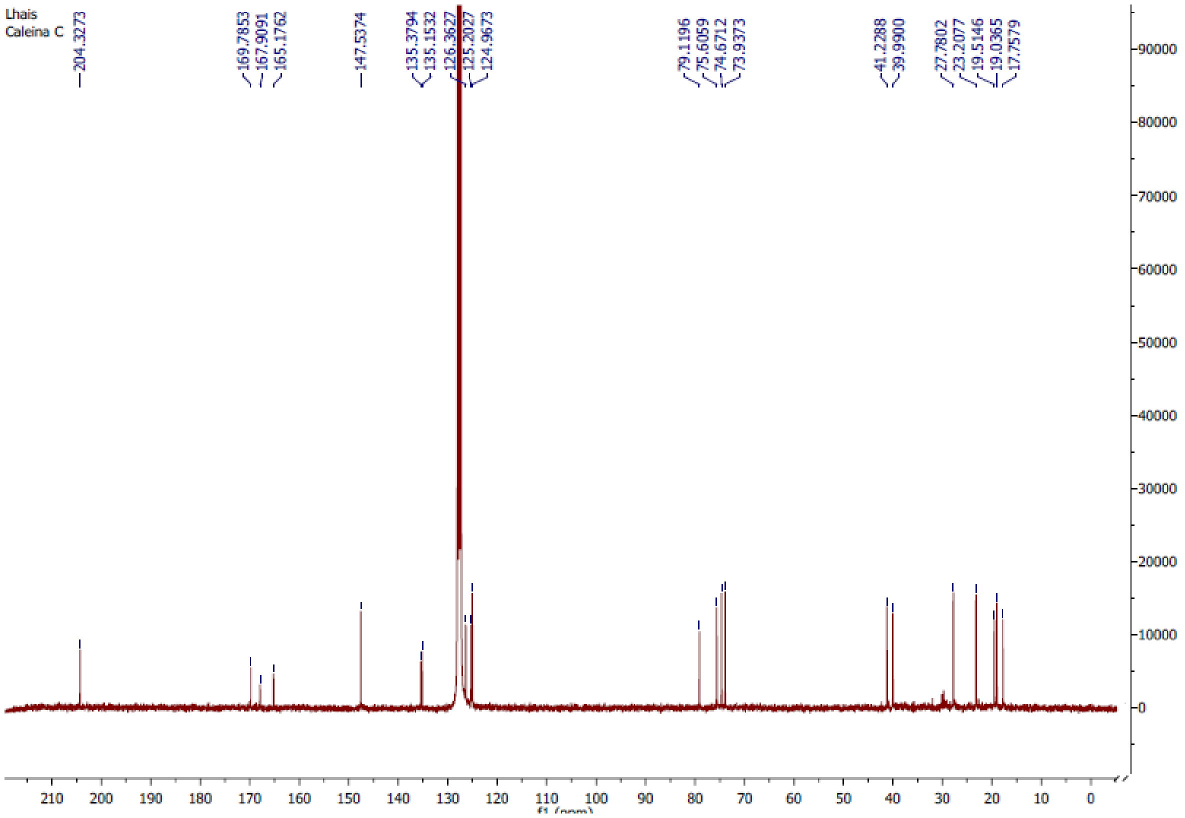

Figure S2: ^{13}C NMR spectrum of **(1)** (75 MHz; Benzene d_6)

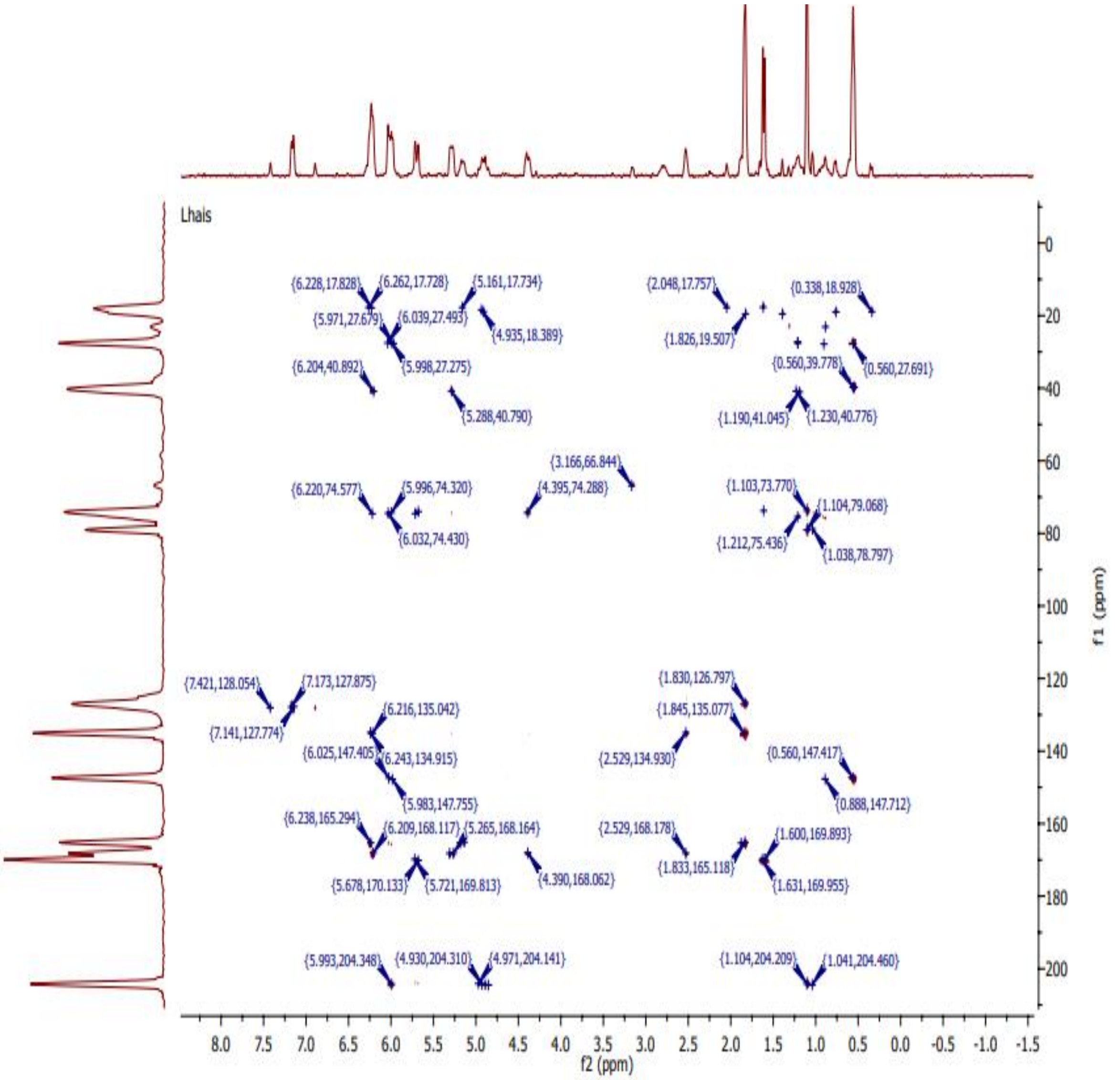

Figure S3: HMBC NMR spectrum of **(1)** (300 MHz; Benzene d_6)

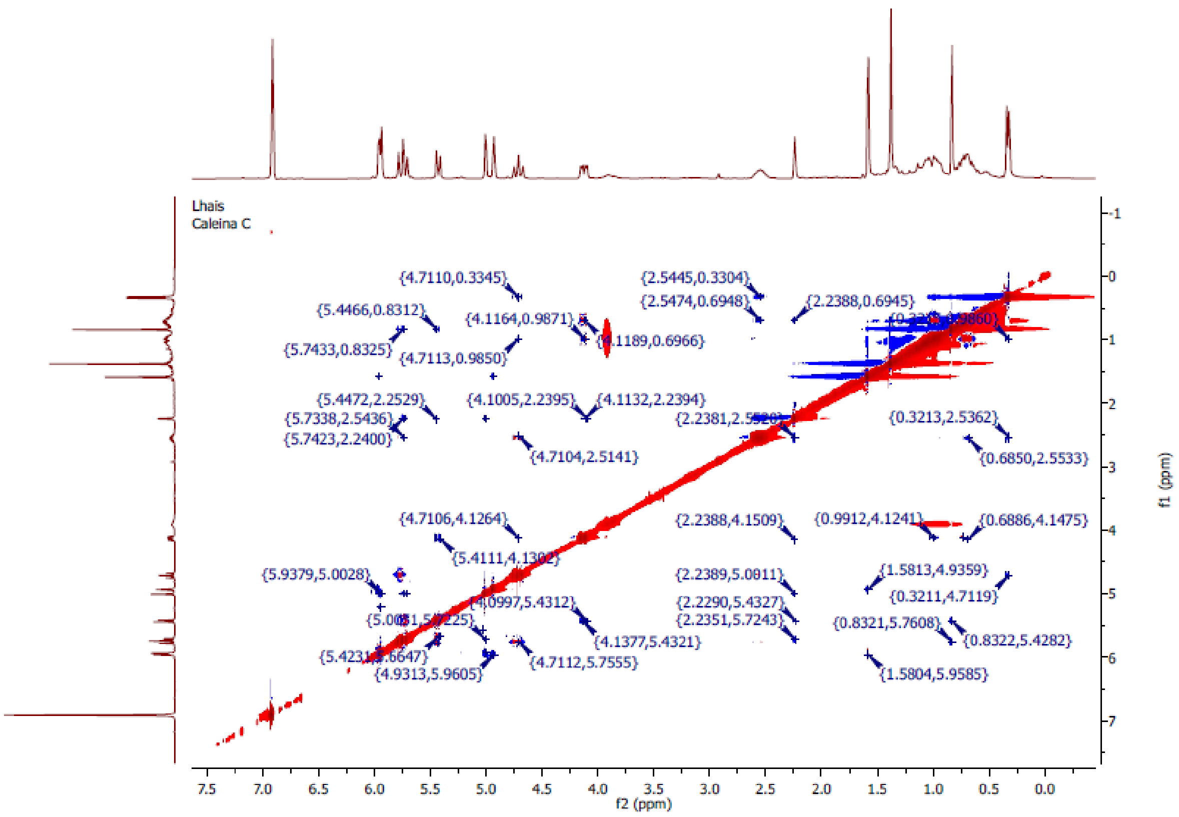

Figure S4: NOESY NMR spectrum of **(1)** (300 MHz; Benzene d_6)

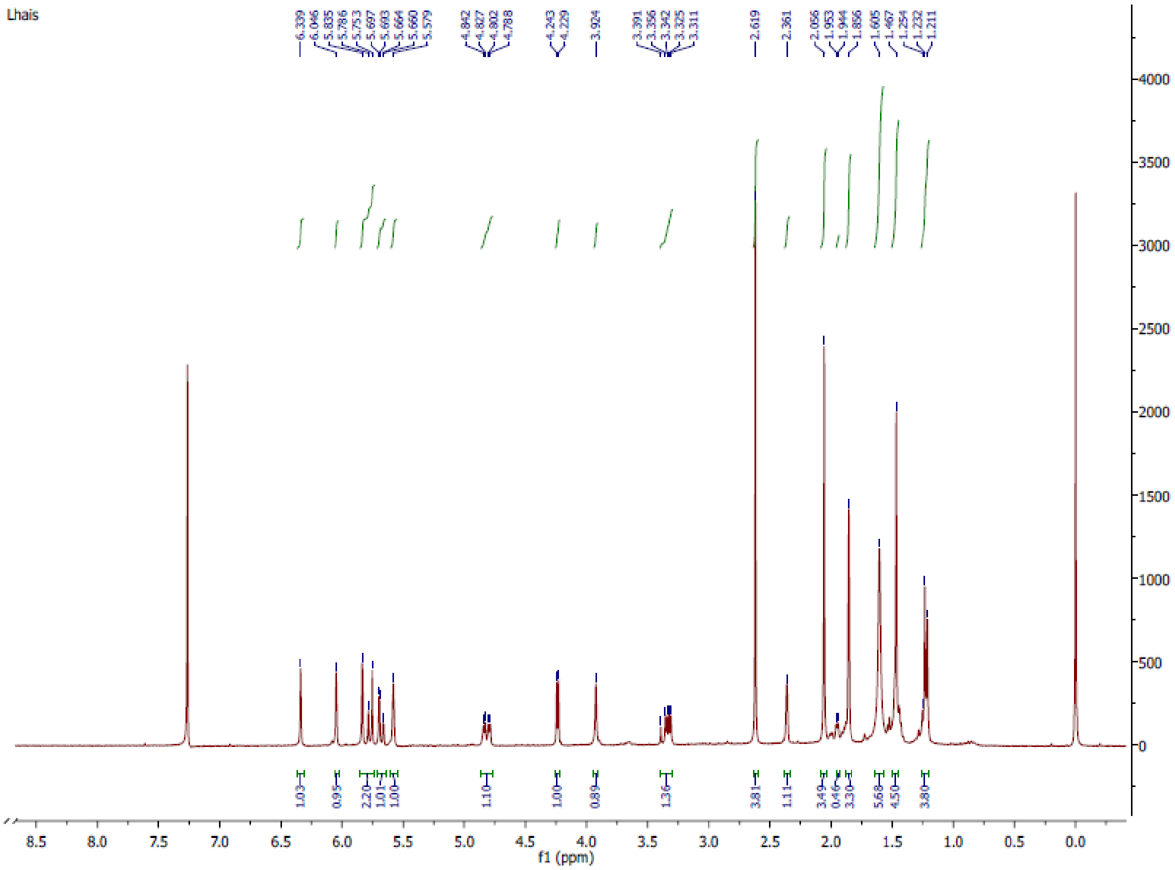

Figure S5: ^{1}H NMR spectrum of **(2)** (300 MHz; $CDCl_3$)

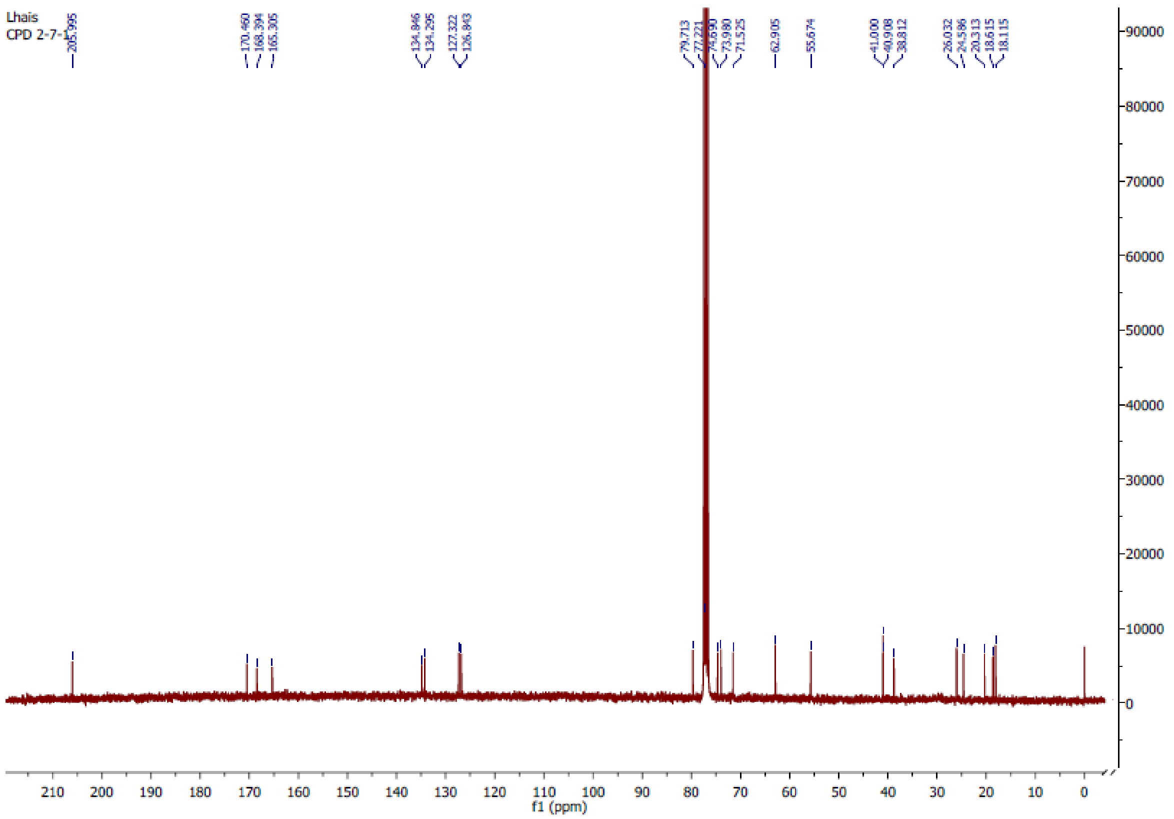

Figure S6: ^{13}C NMR spectrum of **(2)** (75 MHz; $CDCl_3$)

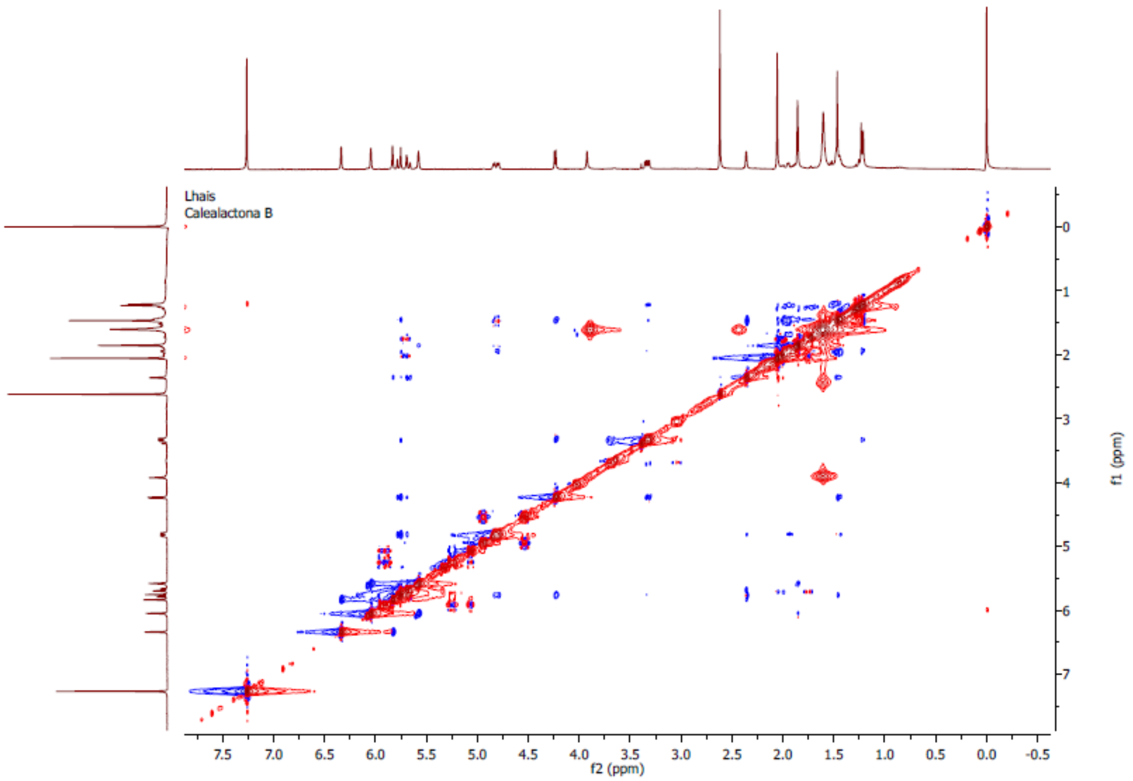

Figure S7: NOESY NMR spectrum of **(2)** (300 MHz; $CDCl_3$)

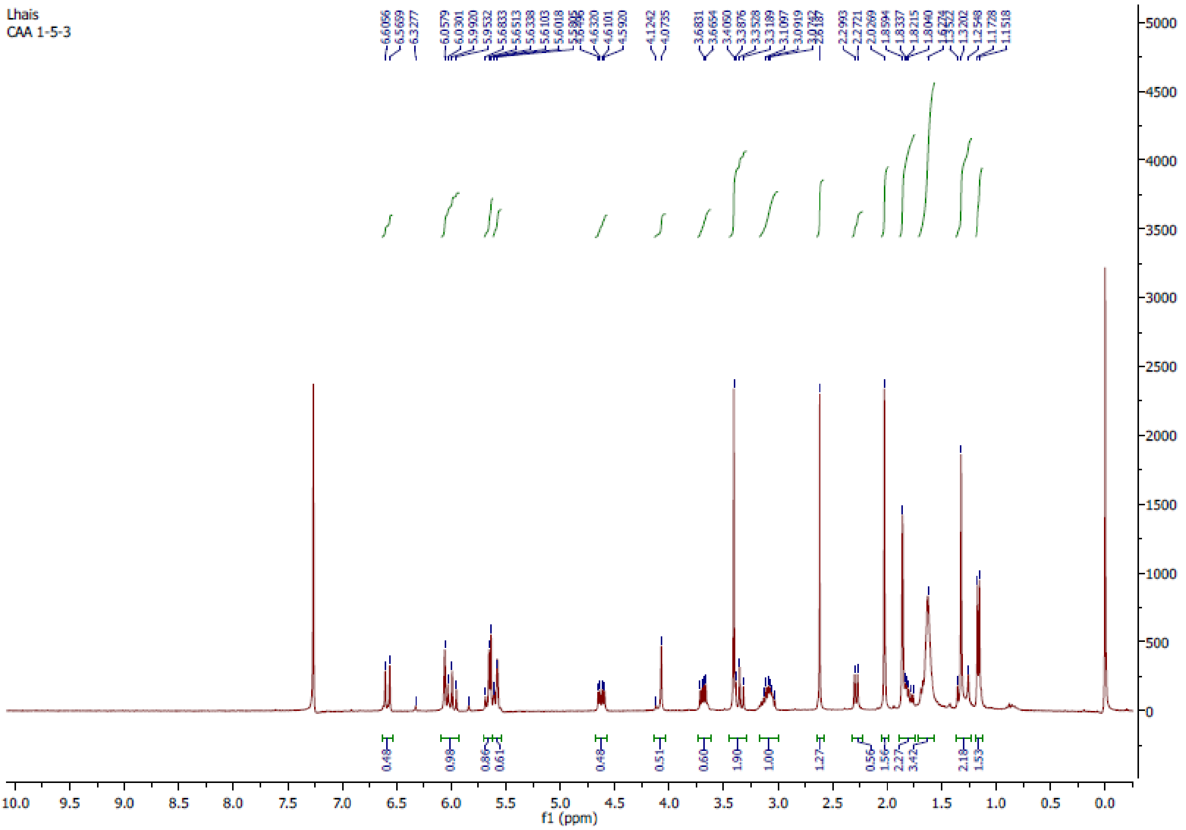

Figure S8: ^{1}H NMR spectrum of **(3)** (300 MHz; $CDCl_3$)

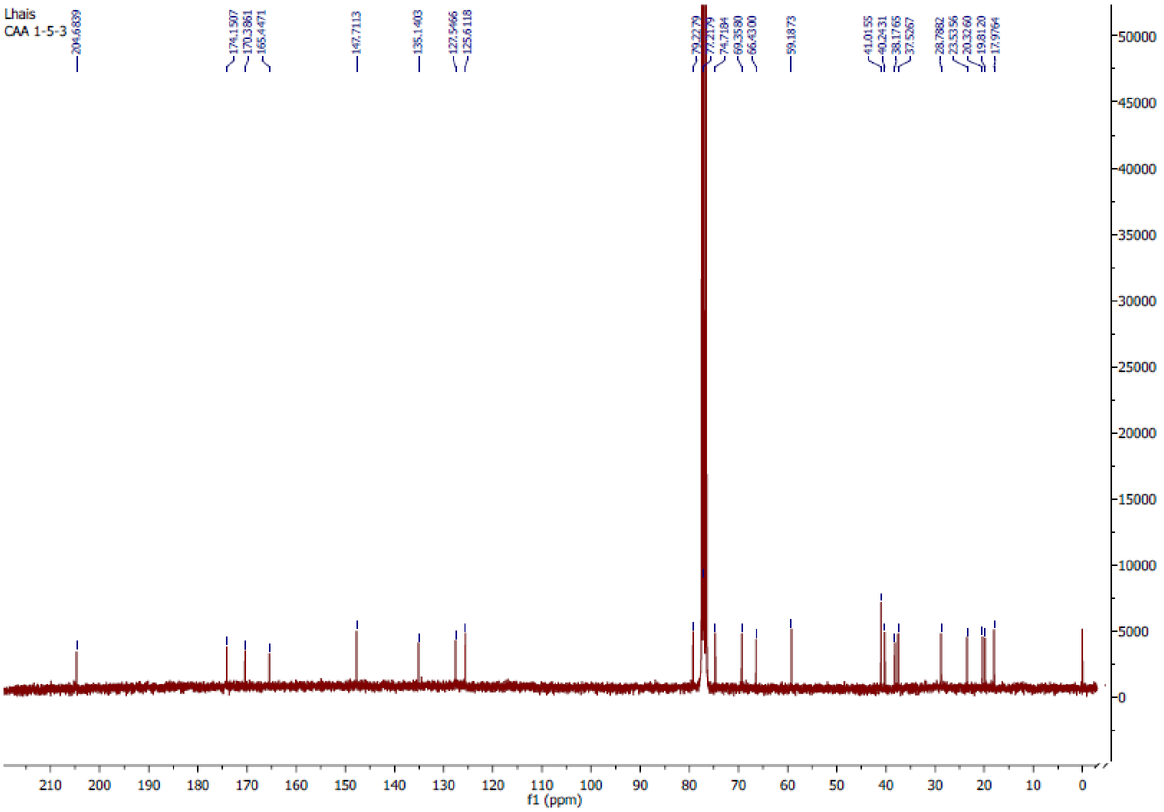

Figure S9: ^{13}C NMR spectrum of **(3)** (75 MHz; $CDCl_3$)
